# Eating Well When Living With an Intellectual Disability—Exploring the Carer: Client Relationship in Residential Settings

**DOI:** 10.1111/jar.70157

**Published:** 2025-11-30

**Authors:** Aslıhan Özdemir, Hazel Chapman, Rebecca Hall, Basma Ellahi

**Affiliations:** ^1^ Department of Nutrition and Dietetics, Faculty of Health Sciences Hacettepe University Ankara Turkey; ^2^ School of Nursing and Healthcare Education University of Chester Chester UK; ^3^ School of Nursing, Midwifery and Social Work University of Salford Salford UK; ^4^ School of Allied and Public Health, Faculty of Health and Social Care University of Chester Chester UK

**Keywords:** carers' influence, healthy eating, intellectual disabilities, nutrition education, person‐centred care

## Abstract

**Background:**

Individuals with intellectual disabilities face increased risks of obesity and health issues. Carers in residential settings play a crucial role in shaping their dietary habits. This study explores how carers influence eating behaviours to identify strategies for healthy eating.

**Method:**

Seventeen semi‐structured interviews were conducted with carers from three community homes. Thematic analysis identified three key themes: (i) whose responsibility is it?; (ii) food autonomy and choice in the context of caring relationships; (iii) opportunities for working together to support dietary choices.

**Results:**

Carers strive to encourage healthy eating while respecting residents' autonomy, particularly in those with cognitive impairments or on psychotropic medications. They use strategies like rapport‐building, personalised care, and nutrition education. However, these efforts are limited by gaps in knowledge, time constraints, and variation in application and impact.

**Conclusions:**

Findings highlight the practice gap and the need for better training and resources to support carers in promoting healthy food choices while respecting residents' autonomy.

## Executive Summary

- Carers face challenges in balancing the promotion of healthy eating whilst respecting residents' autonomy, especially when residents have cognitive impairments or are on psychotropic medications. Strategies like rapport‐building and individualised care are used but vary in effectiveness.
- The findings highlight the critical need for tailored nutritional education and support to enhance the health outcomes of individuals with intellectual disabilities while respecting their autonomy and preferences.
- The study highlights the importance of integrating comprehensive nutritional training for carers and involving nutritionists and dietitians in care planning. This could improve dietary practices in residential settings, ultimately contributing to better health outcomes for this vulnerable population.

## 1 Introduction

Intellectual disability is defined as “a significantly reduced ability to understand new or complex information and to learn and apply new skills (also described as impaired intelligence). This results in a reduced ability to cope independently (impaired social functioning), and begins before adulthood, with a lasting effect on development” (Cluley 2018). Approximately 1.5 million people (2.16% of adults and 2.5% of children) in the UK, are identified as having an intellectual disability (Mencap 2020).

Between 8.1% and 11.5% of adults known to intellectual disabilities services have feeding and swallowing difficulties (Robertson et al. 2017). Ryan et al. (2021) noted that 69% of adults with intellectual disabilities aged 40 or over who took part in the Irish Longitudinal Study on Ageing (IDS‐TILDA) (*n* = 572) were overweight or obese as measured by body mass index (BMI) (Ryan et al. 2021). This compares with approximately 56% of the general adult population of Ireland (Donovan and McNulty 2024). Moreover, adults with intellectual disabilities aged 18–35 are twice as likely to be obese than the general population in the United Kingdom (NHS Digital 2017), with a body mass index of 30 kg/m^2^ or above. Causes of obesity include *primary factors* such as eating habits and physical inactivity, cultural influences, socio‐economic status, and *secondary factors* such as hypothyroidism, psychological or neurological disorders and certain medications including antiepileptic and antipsychotic drugs (Tutor et al. 2023). People with intellectual disabilities experience a greater incidence of epilepsy, severe mental illness, hypothyroidism, obesity, types I and II diabetes, gastro‐oesophageal reflux disease and cardiovascular disease (Perera et al. 2020). Obesity leads to serious illness, including type 2 diabetes, hypertension, pain and reduced mobility (Hsieh et al. 2014), and is linked to the development of chronic health conditions in older people with intellectual disabilities (Ryan et al. 2021). These cumulative health risks contribute to a significantly reduced life expectancy for people with intellectual disabilities, with a median age at death of 66 and 67 years for males and females, respectively, compared with 78.8 years for men and 82.8 years for women in the general UK population (Ryan et al. 2021; Office for National Statistics (ONS) 2025). While these health disparities were not the direct focus of this study, they underscore the critical need for carers to support individuals with intellectual disabilities in adopting healthier lifestyles, which includes diet, since diet is a major modifiable risk factor for many diseases. This study explored the role of carers in shaping dietary habits and addressing any challenges for healthier eating in residential settings, with findings aligning with existing literature that emphasizes the importance of tailored nutritional interventions (Office for National Statistics (ONS) 2025; Spanos, Melville, and Hankey 2013).

In this context, nutritionists and dietitians, professionals who work with clients to improve their nutritional health, play a pivotal role in addressing obesity among people with intellectual disabilities through a multifaceted approach tailored to their unique needs. Education and training of both individuals and their carers on healthy eating practices, including balanced diets, portion control, and calorie intake within recommended values are essential components. Collaborating with carers of clients with an intellectual disability and other healthcare professionals, nutritionists and dietitians implement behavioural strategies to encourage healthy eating patterns while incorporating regular physical activity. These efforts aim to mitigate the risk of obesity‐related complications and improve the overall health and well‐being of individuals with intellectual disabilities (Spanos, Melville, and Hankey 2013; Ptomey and Wittenbrook 2015).

Given the increased risk factors for obesity and related diseases among individuals with intellectual disabilities, there is a heightened need for support in maintaining a healthy lifestyle. However, the ability of individuals with intellectual disabilities to make autonomous dietary choices is often influenced by external factors, such as the structure of residential care environments or the level of support provided by carers (Røstad‐Tollefsen et al. 2025; Boumans et al. 2022). Many individuals report feeling that carers need more training in supporting them to eat well, live well, and manage their weight (Koritsas and Iacono 2016). Effective support requires knowledge of healthy lifestyle support and understanding of the individual needs of the person with an intellectual disability and their family (Nijhof et al. 2024). Dietary care is complicated by ethical issues related to promoting autonomy and choice in people who often need support to make decisions and may think of food, particularly unhealthy food, as rewarding. In some cases, care environments or support systems may unintentionally limit dietary autonomy due to logistical constraints, nutritional policies, or a lack of carer training (Mahon et al. 2019; Pryce et al. 2017). Additionally, food may be perceived as a source of comfort or reward, particularly high‐fat, high‐sugar options, which can further complicate support efforts. Person‐centred care involves empathy and respect (Chapman 2017) as well as engagement, communication, shared decision‐making, a holistic and individualised focus and coordinated care (Eklund et al. 2019). Studies exploring the relationship of people with intellectual disabilities and their consumed diet have generally focused on their health needs, living situation and conditions and dietary habits (Doherty et al. 2018; Willems et al. 2018), but not on the role of the carer. The role of the carer in the client‐carer relationship around food and person‐centred care can be critical to the health of the client. Yet few studies have explored in depth the dynamics of this relationship, the spheres of influence therein and the juxtaposition of person‐centred care and the power relationship (van der Meer et al. 2018; Roeden et al. 2012).

The aim of this study is to examine the dynamics of dietary relationships between carers and people with intellectual disabilities in residential settings. Specifically, it explores how carers perceive their role in supporting positive dietary behaviours and improving the nutritional intake of residents. Rather than identifying common nutritional issues, the study seeks to provide in‐depth insights into carers' lived experiences and the contextual challenges they face. These findings may inform future directions in education, practice, and research to better support carers in promoting nutritional wellbeing within supported living environments.

## 2 Materials and Methods

### 2.1 Research Setting

The study was carried out collaboratively within an independent care sector organisation (a registered charity), which currently provides residential provision for people with intellectual disabilities nationwide, though primarily in the north‐west of England.

### 2.2 Data Collection

Our previous study (Özdemir et al. 2023) used a pragmatist approach (Hall 2013), utilised a mixed‐methods design. This paper focuses on the qualitative aspects which sought to address the research aims outlined earlier. The findings of this phase contribute to a broader exploration of the topic, further developed in Phase 2 through qualitative semi‐structured interviews with carers. This subsequent phase also examined how carers apply their knowledge in context—specifically, how their existing understanding supports everyday food choice decisions for individuals with intellectual disabilities (ID) in residential care settings.

Participants in the study were recruited through a voluntary participation process. All carers employed by the participating organisation were informed about the study during weekly team meetings held at different sites. They were provided with a copy of the participant information sheet and details about the data collection tool. For the qualitative phase of the research, consent was obtained from the third‐sector organisation to approach their community homes. Carers who were on duty at the time of the researchers' visits freely consented to participate in semi‐structured interviews that were guided by a prepared topic guide.

Seventeen semi‐structured one‐to‐one interviews using a topic guide constructed from a review of the literature and findings from phase 1, were undertaken with staff from the care organisation over a three‐month period. All participants in the study were support workers employed by the organisation. All worked in one of three small community homes located in different geographical areas.

The community homes each provide individual home‐based accommodation for up to eight people with an intellectual disability. The residents have a variety of needs ranging from care and support with all activities of living, to those who have some independence and can access their local community more independently. Promoting choice, engaging with the local community and supporting the development of new skills are fundamental principles of care provided through the homes.

Carers had varying lengths of experience in care, ranging from 1 month to 23 years. Recruitment included staff from homes supporting clients with diverse levels of ability, from individuals living independently in self‐contained flats to those requiring full‐time care, including percutaneous endoscopic gastrostomy (PEG)‐fed residents, clients with dysphagia needing liquidised or soft foods, and those entirely dependent on staff for their needs. Recruitment was conducted through direct engagement with care homes; participants represented a range of roles and experience levels within the organisation. In relation to training in nutrition previously received, all staff completed mandatory training every 2 years, which included a brief refresher on food hygiene and nutrition. The insufficiency of this training is picked up in the one‐to‐one carer interviews and will be discussed later. Two team leaders had additional qualifications (NVQ Levels 2 and 3 in Health and Social Care) but reported that these qualifications did not include detailed coverage of nutrition. The homes also reported variability in client needs, including managing dietary requirements for residents with complex conditions, such as dysphagia, and accommodating preferences for those who were reluctant to try new foods.

### 2.3 Ethics

Ethical approval was obtained from the Faculty Research Ethics Sub‐Committee at the University of Chester. Participation was voluntary and all participants were provided with an information sheet explaining research aims, the interview process, how data would be treated, anonymity and confidentiality issues. All participants signed a consent form prior to participating. Interviews were run by three investigators, audio‐recorded, transcribed and assigned a pseudonym to maintain anonymity. The data were handled in compliance with the General Data Protection Regulation (GDPR) (2018).

### 2.4 Data Analysis

Interviews were transcribed verbatim and checked with original recordings by research team members. Thematic analysis of the interview transcripts (following Braun and Clarke 2006) (Braun and Clarke 2006) was undertaken. Data analysis followed the six phases of thematic analysis, whereby comprehensive immersion in the transcripts was undertaken which were read and re‐read to generate initial codes. The process is resolved with analysis in the context of the literature and referral back to the original transcripts where necessary. The qualitative software package NVivo (12 Plus) (QSR International Pty Ltd 2018) was employed to assist the analysis, particularly useful in the storage and subsequent organization of the data and facilitating the necessary structure to enhance rigorous analysis and checking by co‐researchers. Agreement of themes and sub‐themes was obtained through the third researcher who helped refine these until consensus was reached using a fourth researcher.

Common themes were collated and selections of text which largely expressed the majority viewpoint or consensus were used. Where possible and data permitted differences between views are reported as were any deviations. Quotes used are anonymized, using only the pseudonym as identifiers. Person X used in the text, refers to clients the interviewees referred to.

### 2.5 Rigour

Methodological rigour was assured by Consolidated Criteria for Reporting Qualitative Studies (COREQ) (Booth et al. 2014). For qualitative analysis two researchers were involved in interview data analysis to generate codes prior to the production of the final themes, guard against researcher bias and enhance the validity of the coding method. Rigour, particularly authenticity, was addressed by a process of constantly cross‐checking interpretations with the original transcripts, which ensured participant voices were heard (Ajjawi and Higgs 2007) and enhanced faithfulness to their interpretations (Lincoln and Guba 2000). Continuing inter‐researcher dialogue, furthermore, served to facilitate reflection, consider alternative interpretations, consolidate insights and ensure thorough interrogation of the data (Barbour 2001). The constant consideration of such criteria throughout the research process promoted the necessary ‘methodological awareness' (Seale 2002) for rigorous engagement with the data (Barusch et al. 2011).

## 3 Results and Discussion

Seventeen employed carers participated in interviews for this study which explored their experiences, insights and perspectives on the dietary choices and habits of service users with intellectual disabilities. The residential homes supported a range of client needs. Some clients required full‐time care, whilst others were more independent living in self‐contained flats situated within the same building. All support workers maintained records for each resident and logged activities including food consumed to monitor dietary intake. Clients were encouraged to accompany support staff on shopping trips where the staff, member can assist with the selection of foods according to a pre‐planned shopping list. In general clients did not have freely open access to the kitchen, due to safety reasons and managing the food costs; however, clients were encouraged to help carers with the preparation of meals. The exception to this was where clients were in their own flats. These support workers also assisted clients living in self‐contained flats to plan, budget and prepare meals.

The findings revealed significant insights into the practices used by carers and their impact on the eating habits of individuals with intellectual disabilities. People living with intellectual disabilities face difficulties such as short‐term memory impairment, difficulty in grasping concepts, problem‐solving skills, and understanding the consequences of their behaviour (Røstad‐Tollefsen et al. 2021). Although nutritional knowledge is not a prerequisite for carers, it is recognised that adults living in residential homes are prone to weight disturbances and malnutrition (Ptomey and Wittenbrook 2015). Specific factors such as having Down Syndrome or being on certain types of medication (such as psychotropic medication) increase the risk of obesity (Ranjan et al. 2018). Carers have the potential to influence lifestyle and behaviour choices, leading to positive outcomes such as increased physical activity, improved routines, and quality of life (Lynch et al. 2022; George et al. 2020). However, there may be a lack of clarity when it comes to reducing weight and improving health through healthier eating habits (Elinder et al. 2018).

The initial stage of theme construction was undertaken by two researchers (a nutritionist and a dietitian). A third researcher contributed critical insights from a nursing and caring perspective, while a fourth researcher provided an intellectual disabilities (ID) perspective that was instrumental in shaping the development of sub‐themes and final themes.

Three themes were developed from the data as a consequence of some of the tensions evident in the narratives. For example, while accepting that part of their role was to support clients to make healthy food choices while shopping and preparing food, carers also identified some ethical and practical barriers to achieving this. A further contradiction was between physical health and emotional and behavioural wellbeing, where food was found to be used as a short‐term reward. Some resolution was found in the third theme, where some participants identified the importance of engaging the person with intellectual disabilities in choosing ingredients and preparing their own, healthier, food. Although this last theme was less evident in the data, it was included due to its importance in suggesting some potential interventions for carers to enhance nutritional support for clients in a supportive approach. Thus, the final themes were: (i) *whose responsibility is it?* (ii) *food autonomy and choice in the context of caring relationships* and (iii) *opportunities for working together to support dietary choices*. The theme development process is presented in Table S1, and the final themes and sub‐themes, along with illustrative quotations, are presented in Table 1.

- Whose responsibility is it?

**TABLE 1:** Themes and sub‐themes with illustrative quotations from carers of people with intellectual disabilities.

Themes and sub‐themes	Quotations from carers
1. Whose responsibility is it?	
Sub‐theme 1: On‐going dialogue to influence and guide around choices	“We suggest that they could have this instead of that and with Client X—‘it's got a lower calorie count in that one’ and she will take that on board. She won't always swop it. Sometimes she will put both in because she thinks that is what you want her to do. So you have got to be kind of very careful with the wording. Again, you kind of try and encourage and say ‘oh that one's a little bit healthier for you’—‘if we get this it is a little bit healthier for you’—‘you know you are trying to lose a little bit of weight, well this will help you to lose that little bit of weight’ – so you have got to be kind of very soft with Client X because if you say ‘you are not getting that’ or anything like that, she will get it.” (Olivia) (not reported in the text) “It is just the way you talk to people. You have got to get to know them—that's the key is getting to know the person and how to talk to that particular person. Because you talk to Client X totally different to the way you talk to Client Y when you are advising her. You can be very straight and very blunt with Client Y but with Client X you have got to kind of soft‐soap it a little bit more and say it in a different way but if you didn't know either of them you wouldn't know that you needed to do that.” (Lyndsie)
Sub‐theme 2: Delivering the personal plan for high dependency adults	“…we have …, a personal plan, where all the guys have a folder each about them, about their food, their dislikes, their likes and we write what they have done in it. So we have like a Monday, like the food chart, we have a chart for activities and stuff, so we write what they have done for the day and then we have a food chart, like what they have had for their dinner/tea. We have got to do that every day, so they can keep on track. Then we can have a look back, so if they have had beans on toast 1 day, we don't do it the next day, we have just got to keep an eye on it, because sometimes you can, if you don't look, you can always cook something the same and you get a bit bored if you have it all the time don't you?” (Penny) (not provided in the text)
Sub‐theme 3: Responsibility and duty of care to ensure a healthy diet?	“I would say theirs and ours. We have got both… It predominantly lies with them because they have got the capacity in this service but I definitely think we have a responsibility to kind of try and influence and advise them as best we can to have a healthy diet. Again, you can only have so much of an influence and advise so much but… I definitely think it works both ways. If they know they can come to us and ask us for a bit of advice, which they do, then I think we can kind of try and work together early on and try to get the balance right.” (Olivia) (not provided in the text) “It is everybody's responsibility. It is all of our responsibility from the manager, from my regional director who is my manager, to me, the whole organisation, GPs, the district nurses, it is everybody's responsibility. I suppose it is the people who work on the floor. It is the support workers who have to implement…It is them who have to put it into place. It is everybody's responsibility but it wouldn't happen if it wasn't for the support workers.” (Colin) (not provided in the text) “I would say the staff because we are the ones that are supporting them 24/7 and as I say, if they can't speak then it is up to us to try and make…… I was going to say make decisions for them but not that I am actually making decisions for them, to be honest. As I say, as long as you are buying stuff that they like and still getting the goodies, but moderate, do you know what I mean. Not just ‘what do you want to eat today, you can either have that or you can have a bar of chocolate’. As I say, it is more for like suppers or sitting and watching the telly or something like that—watching a film.” (Gail) (not provided in the text) “All the staff members. The whole staff team and the Manager as well because we have got a Duty of Care to maintain their health and wellbeing and to try, as I say, to provide as healthy a diet as possible to ensure that they stay healthy.” (Nicola) (not provided in the text)
Sub‐theme 4: Respecting clients' choices	“That's their choice and there is nothing we can do other than say ‘you have been out this week, you have bought your snacks for the week and when they are gone they are gone’. If it is gone in a day well that is up to them. Yes, we do have issues with a lot of overeating at the other place. There is nothing really that we can do because in their best interests you can go to a dietician who will advise try this, this and this and we can go ‘yes OK’ but we can't force them.” (Helen) (not provided in the text) “There is another woman who… She is sensible, you know. She will come in you will see. She is very sensible and then there is one who has got a bit of a problem but we have really worked hard on her to get her balanced, but it is really quite hard because we can't control it when she is going to the shop. We can't tell her not to get a chocolate bar and that, but we have put some real good effort into her but it is hard obviously because we haven't got control.” (Malcolm) (not provided in the text) “…we have to leave him a choice and we also discuss with him and ask if he is willing to bargain with us if you like. So, we don't take the things away from him, we just try and encourage him in a smaller proportion, or maybe don't leave him to his own devices. Offer him an opportunity to store—he likes to buy chocolate bars for example when he walks off to the shop independently. That is just something he does, we can't stop, we wouldn't want to stop.” (Gary) “That's their choice and there is nothing we can do other than say ‘you have been out this week, you have bought your snacks for the week and when they are gone they are gone’. If it is gone in a day well that is up to them. Yes, we do have issues with a lot of overeating at the other place. There is nothing really that we can do because in their best interests you can go to a dietician who will advise try this, this and this and we can go ‘yes OK’ but we can't force them.” (Jenny)
Sub‐theme 5: Blurred boundaries‐Challenges of advice and enforcement (consideration of external life factors)	“All we can do is our best which is try and influence them. We cannot force them” (Barbara) (not provided in the text). “I know people who have been that way inclined and said it is their life they can have what they want but when you know it is going to affect them majorly, because I have seen some people‘s weight flux, so I will put that kind of restriction in place for them because they haven‘t got the mental capacity to. I do believe that restrictions sometimes need to be put in place for their own benefit and health.” (Alison) (not provided in the text) “There‘s not much you really can do. Like I say if they want chips you can guide them to the sweet potato chips. If they want chips well it is ‘OK what about if you just have a few then, not quite all of them just have a few?’ At the end of the day we are not here to play God, we are not here to say ‘no’, but it is about having best interests. From my point of view as well advise all you can but at the end of the day” (Helen) (not provided in the text) “It is my responsibility to promote what‘s you know healthy things to them, but it is not my decision, it‘s not mine. It‘s their choice. It whether they you know in most ways they decide they don‘t want to … because we all think we know best don‘t we?” (Frank) (not provided in the text) “If they want a cake they can have a cake but we would talk to them and try to come to some agreement or understanding like you can have one a day or maybe…. yes. It is a hard one that because, as you say, it is their choice at the end of the day if they want to sit here and eat bags and bags and bags of chips then that is up to them. The only thing we can do is just try and cut it down a wee bit, but as I say at the end of the day it is their choice. It is like somebody telling me I couldn‘t drink Coke—at the end of the day that is my choice.” (Gail) (not provided in the text) “You don‘t realise how big a part food and drink plays in the job that we do but the amount of time that we do actually spend preparing, thinking about, monitoring it, recording what they all eat in a day—yes, it is quite big.” (Helen) (not provided in the text) “…we are not God, we are not here to say yes and no but at the same time we are not here to let them eat and drink themselves to death or starve themselves or dehydrate themselves—it is a fine line but it is finding that compromise.” (Helen) (not provided in the text)
	“…it's their choice at the end of the day and I mean your biggest part of being a support worker is making sure other people have their choices and that it is all about them, you know and diversity and all the rest of the work done. You know I know if I am sitting at home and I feel like having a cup of tea with a biscuit, well why not? So why, because they have got learning disabilities should they be any different” (Kerry) (not provided in the text) “…we try and encourage as much as we can and tell them the benefits of eating healthily against the benefits of not eating healthily but again, we can't force them into doing anything. You can't force them into eating a meal when they are out… We can't force them into A or B. We can't have that influence over them. Again, we can advise them but we can't say ‘you are not having that because it is unhealthy’. We are not allowed to do that.” (Lyndsie)
2. Food autonomy and choice in the context of caring relationships	
Sub‐theme 1: Food as a reward	“I don't like it at all. I don't think it is good because then that becomes another manifestation on…. ‘you cleaned your room, here is a chocolate bar’. Then they will go off ‘I've cleaned my room where is my chocolate bar?’ I believe when someone has had a good week then you go out and celebrate and go bowling and then go out for dinner and you kind of make it look more of an activity end than food because if someone starts thinking that ‘I do this I get food’ then they will do more of it. I don't believe in it really. I don't want to sound blunt here but it is like a dog, you know. You do good you get a treat and I just don't like that type of……and I try and make it more of an activity if they want rewarding then they say ‘well because of all this great stuff then we are going to go out and do this’. I try and stay away from that because I have seen that before and then it is just expected and expected and expected or if they do a good thing in this—‘where is this?’ That leads again onto challenging behaviour because they aren't getting what they want so I think with it, with reinforcement, I just think if they do something good then you make it into an activity or a pamper day or something. I just think there are ways around where you don't have to use food. Client X would never do anything on a Sunday—oh we struggled with that! He would just sit in his lounge and watch telly all day and do nothing. It used to bug me so I would say ‘right come on then let's go for a walk around South Park’ and then he would be like ‘where's the food?’ Do you know what I mean—where is the food involved? So in the end with him I used to take him for an hour round South Park. I guess if you reinforce exercise then it is not much then so I guess it's sold more likely on what he is going to benefit.” (Alison) (not provided in the text) “I don't think that is right. If they want a reward what about…. What do they like? A day at Alton Towers, you know, walk round West Kirby, go round Cheshire Oaks.” (Barbara) (not provided in the text) “That is just control. That's not a reward that is somebody trying to control somebody.” (Gail) (not provided in the text) “I can't say anything I have had a fat dog for 9 years! I am not big on it at all because I think that is just… in fact I am right against it. I'd rather do something else. There's other ways of reinforcing positive behaviour—not bribing someone. That's how I see it. If you are going to bribe them then what else are you going to bribe someone with; so I'm sorry it is a no‐go in my…” (Helen) (not provided in the text)
	“Sometimes to stop challenging behaviour you would give whatever they wanted, then a lot of people in learning disability, for some reason it is all around food. A lot of people I have worked with have said it is challenging around food and drinks. If someone is going to be challenging towards you or possibly hurt you or you can go ‘let's go McDonald's’. As a support worker you weigh that up and the McDonald's always won. Changes wise—we do try and stop that now. You know people have got more robust planners in place to say ‘this is their treat day, this is their meal day’ no more. That's kind of specific in what I do in one of my other services—all the challenging behaviour is around food—McDonald's, KFC, the pub. It is all around that and he will ask you for them all day every day. He has 1 day on a Tuesday now and then 1 day on a Friday whereas we used to go ‘just let him have it’. It's what he wants. He is making that choice, but now we do promote the fact that… We are paid to deal with challenging behaviour so let's… healthy diets. So we do promote that more whereas 10 years I know for a fact that I never used to promote any sort of…. I just never.” (Colin) (not provided in the text) “I wouldn't say I was against it, because if you have got somebody who is having severe behavioural issues and… it would depend on the situation. I would have to say it would depend on the situation. You know I wouldn't say to them ‘right if you don't eat this, you're not getting that’! That is totally unacceptable, but if you are trying to calm somebody down that's in a manic state, I suppose I would say yes I would offer it. Obviously not all the time but it would depend on the situation. You know if you knew that given somebody something was going to bring them down, and make them and you know their behaviours and them calmer, and would do them more good, than them like bashing their head against the wall, or trying to smash the place up, then yeah I would say yeah.” (Lorraine) (not provided in the text) “That won't work at all because if you start rewarding somebody with food you are just going to ruin their diet. Instead of saying ‘maybe I'll give you some chocolate’ say ‘maybe I will take you swimming’, something they will enjoy. I don't think food should be used as a reward in any circumstances.” (Lee)
Sub‐theme 2: Less dependency	“We do keep a record sometimes of what they have eaten but as I say, sometimes you don't know what they are eating because they just run out of the room with a bag,” (Malcolm) (not provided in the text) “…you do have the communication book as well here. So, you know where we will write down you know so‐and‐so hasn't eaten very well today, please can you ensure … or so‐and‐so hasn't drank much, can you please push fluids and ensure… So it is good, it is a 24 h…” (Lorraine) (not provided in the text) “…we can't keep records of what the other guys are eating. We wouldn't do it at home and that's what we are trying to encourage here is their independence as much as possible. I know we are watching but we are not in a way as well so we try and encourage their independence as much as possible so to get them into a routine of having breakfast, lunch, dinner, or having three meals a day and remembering to have three meals a day without us prompting them, because we are not obviously going to be there when they move out…” (Olivia) (not provided in the text)
Sub‐theme 3: Families as external influences on eating—challanges/barriers (and opportunities)	“His Dad used to bring him food and in the end we sat down and said this can't keep going on because 1 day he came back with this big bag of M and Ms saying he didn't have chance to eat them at the day centre. We were like well we didn't even know you were getting these M and Ms. He was overweight himself. The hardest part is speaking with the families because we had one client, Z—I know I shouldn't be telling you names His parents had quite an emotional thing because they felt sorry that they couldn't look after him and they knew Hugh loved food and so on a Sunday they would bring him bags and bags and he was getting bigger and bigger.” (Alison) (not provided in the text) “…one lady where we used to work, there was one lady that lived on the other unit of our house, who used to see her family at the weekend and she knew that once Saturday came she got chocolate and that was all that she would do get the chocolate and she would go again. She wasn't really interested in the family, she associated family and chocolate and that was her thing, she didn't really have the relationship with her mum and dad, it was with the food. Which to me isn't good, it's you know I think what they have done in the past is they have used that as an incentive for her to see her family and I personally think that that is wrong. Rather than building up…” (Lisa)
3. Opportunities for working together to support dietary choices	
Sub‐theme 1: Positive reinforcement and practices for healthy behaviours —Praising around healthy behaviours	…“We try and encourage the tenants where we can. It is not always successful, but we try our best to encourage and promote it really.” (not provided in the text) “She likes a lot of praise—so the more you praise her if she is eating healthily, and she is losing the weight…the more you praise her and… We have given her certificates that we have printed off and just mocked up on the computer very quickly. She loves that kind of thing because she just loves the praise that she has done something that was good because historically in her childhood she never got that. She never got any praise for doing anything good.” (Lyndsie)
Sub‐theme 2: Negotiations around behavioural choices	“We go along to Weightwatchers with her or Slimming World with her and encourage her as best we can to be cooking from fresh. She will still go down to the shops and she will still buy a ready meal or something like that. We can't stop her. We can only advise her—‘you don't want to do that—let's cook it fresh instead—let's go and get the fresh ingredients, you will find it is just as cheap as £3 for a ready meal—we'll go to Aldi, it is only around the corner.’” (Olivia) (not provided in the text)
Sub‐theme 3: Providing advice	“You could advise them to get other stuff and remove some chocolate bars but your hands are always tied by laws and regulations. You can't say… Say, for example, I went ‘you are not having any chocolate bars, put them back’—I would be breaking the law. So all you can do is give them the best advice you can and they listen to you because they know you are there for their own good. If they don't all you can do is advise healthy food for them.” (Malcolm) (not provided in the text)

Carers in this study acknowledged the challenges in relation to their responsibility, associated with encouraging adequate nutrition and food consumption. This included time constraints, knowledge gaps, communication difficulties, and the tension between autonomy and promoting healthy choices. Building rapport, adopting an individualised approach, and understanding preferences were seen as important factors for effective communication and positive choice outcomes:

…the key is getting to know the person and how to talk to that particular person

In our study, carers were mindful of the need to respect clients' choices, but they often felt unable to influence poor food choices restricted by their roles:

We can't force them into A or B…we can advise them, but we can't say “you are not having that because it is unhealthy”. We are not allowed to do that”

Similar experiences were identified in a study conducted in the UK, where homes housing residents with higher intellectual abilities in a less restrictive setting were more likely to be associated with a poor diet and a higher incidence of obesity (Robertson et al. 2017).

In the past, decisions related to diet and other aspects of life were made for individuals with intellectual disabilities. Significant efforts have been made to address this, as choice is a strong determinant of quality of life (Dover and Lambert 2016; Wehmeyer 2020). However, the availability and attractiveness of healthy foods are essential for promoting a healthy diet (Monterrosa et al. 2020). Facilitating choice, especially if people with intellectual disabilities make unhealthy food choices, must be balanced by the professional duty of care. An exclusive focus on protecting civil liberties, coupled with the potential consequences of misjudging an individual's ability to understand the impact of their choices could result in harm (Smyth and Bell 2006). However, there are instances where some “treats” form the basis for healthy social interaction with the community and may be acceptable within a balanced diet:

…he likes to buy chocolate bars for example when he walks off to the shop independently. That is just something he does, we can't stop, we wouldn't want to stop.

The carers participating in the study felt a sense of responsibility to encourage and promote healthy lifestyles. They also recognised that this can be particularly challenging with residents who have more autonomy and independent access to shops. One carer used the term ‘bargaining’ to describe interactions with clients aimed at reducing portion sizes and the volume of snacks purchased. It was acknowledged that this approach was not always successful, and that staff had to accept the clients' right to choice. The dichotomy between facilitating quality of life in relation to personal choice and fulfilling the responsibility of duty of care is a complex issue that can often go unnoticed. Thus, it emphasises the difficulty of determining when a choice made by individuals with intellectual disabilities is truly understood (Carney and Beaupert 2013).

Despite having the best intentions, the concept of individual choice within a residential setting is often constrained to the availability of food and pre‐determined options (Hollomotz 2014). Carers have acknowledged that meal choices and snacks for the residents are influenced by their own knowledge, skills, and preferences (Røstad‐Tollefsen et al. 2021). In this study, one carer acknowledged:

…some people just don't know how to cook. Some of the staff don't have brilliant diets….Pot Noodle and a coffee…

In most cases, meal planning was used to ensure variety and prevent repetition, not only for residents who are more dependent but also for those who can make decisions for themselves. However, it is evident that practical considerations such as time and cost come into play:

If you have got a really busy day.… ahead….then you just go and slam in pizza and chips…

adding to the challenges and tensions between promoting health, autonomy, and healthy choices (Gjermestad et al. 2017). Other staff, however, identified interest in and knowledge around nutrition:

…you have your standard health…. training…. I have been to nutritionists…. [and]I love health documentaries…

The importance of having access to dietitians and speech and language therapists for advice and more detailed information about diet was welcomed by carers in this study:

… the dietitians here are great… And the SALT (Speech and Language Therapy) team…. they are always on the end of an email, or you can ring up for advice…. They teach you more than what our training teaches…

Recognising the importance of exercising freedom of choice in daily living, it is crucial to understand that not all adults have the capacity to fully consider the health implications of their choices (Scott and Havercamp 2016). In accordance with the Convention on the Rights of Persons with Disabilities (CRPD, 2007), (Rasmussen and Lewis 2007) individuals requiring more intensive support should still be afforded every opportunity to exercise autonomy and be encouraged in their day‐to‐day lives. Carers acknowledged the challenge of limiting or removing choices when residents desire to consume unhealthy foods, highlighting the delicate balance between fulfilling their duty of care and respecting individuals' autonomy:

It is the only pleasure they get through the day….is what they are going to eat at mealtimes.

In our study, whenever possible, carers actively encouraged residents to participate in menu selection and create a shopping list. Autonomy and choice were seen as essential human rights:

He has got lack of capacity in a lot of areas of his life but that doesn't mean he has got lack of capacity in what he wants to eat…

While accompanying residents to the grocery store, carers tried to enhance awareness regarding the widespread availability and marketing of less healthy foods. They assisted in examining food labels and promoting the substitution of unhealthy items with healthier alternatives:

I always say to them ‘look at all the fat in that – oh look at all that sugar!’ The likes of Person X wouldn't know what the hell you were talking about with the amber traffic lights.

However, the effectiveness of these efforts may vary depending on the knowledge and preferences of the carers (Røstad‐Tollefsen et al. 2021). Additionally, food labelling can potentially be confusing for individuals with intellectual disabilities (Doherty et al. 2020).

During the study, residents were not restricted from making unhealthy choices, as the interventions underscored the significance of respecting and facilitating participation based on their own will and preferences. Nonetheless, implementing supported decision‐making can pose greater challenges for individuals with more severe intellectual disabilities (Bigby et al. 2022). Carers explained that personal plans detailing residents' likes and dislikes, as well as documenting their activities and food consumption, are crucial aspects of care:

… it is very important with the foods to make sure their religious beliefs and their rights and past trends and histories are referred to. Just because people can't speak…. If they are all in an age group, then it is kind of do your best guess that on a Friday they would eat fish; do your best guess on Easter Sunday they would have turkey…. It is about the traditional eating as well.

Carers responsible for assisting adults with intellectual disabilities in the UK face the challenge of balancing two potentially conflicting duties: protecting service users (duty of care) and respecting their autonomy (Hawkins et al. 2011). Autonomy implies that individuals have control over their lives and make decisions based on their own preferences, values, and beliefs. In this study, carers in group homes maintain records for each resident, logging activities and food consumed to monitor dietary intake and prevent repetition. This practice applies to both dependent and more independent clients in the homes.

ii Food autonomy and choice in the context of caring relationships

Previous studies have shown that using food as a reward for good behaviour was a common approach in individuals with intellectual disabilities (Spanos, Hankey, et al. 2013; Cartwright et al. 2015). However, in our study, several participants expressed concerns about this practice, as it can create an unhealthy association between good behaviour and food, potentially leading to unhealthy eating habits:

….she…used to see her family at the weekend and she knew that once Saturday came she got chocolate and that was all that she would do get the chocolate and she would go again. She … associated family and chocolate

Children, adolescents, and individuals with intellectual disabilities share common tendencies for challenging behaviours (Allen 2008; Wolkorte et al. 2019; Tevis and Matson 2022) and their reliance on parents or carers, resulting in similar behavioural approaches by carers and parents. Using food as a reward for challenging behaviours is analogous to parents offering their children a favourite snack to encourage them to eat their vegetables (Carr and Epstein 2020), but it is not a sustainable strategy. Furthermore, carers preferred to view their clients as individuals with needs and preferences, rather than solely focusing on compliance. Some carers perceived the use of food as a reward as a form of control and instead suggested alternative activities or experiences as rewards. However, they also recognised that in certain situations, such as managing severe challenging behaviour, food may be a necessary tool to help calm an individual down. Ultimately, there was a consensus among the participants that food should not be the default or preferred reward, and that there are other ways to reinforce positive behaviour:

Instead of saying ‘maybe I'll give you some chocolate’ say ‘maybe I will take you swimming’, something they will enjoy. I don't think food should be used as a reward in any circumstances.

Carers emphasised the importance of striking a balance between monitoring clients' health and nutrition and promoting their independence and autonomy. A fundamental aspect of competence is the ability to exert control over the amount, types, timing, and location of food consumption, as is expected in the life of a healthy adult. Individuals with intellectual disabilities often struggle with controlling their food intake and exhibit eating habits resembling those of childhood. As a result, conflicts over nutrition and healthcare occasionally arise between individuals with intellectual disabilities and their carers (Davies 2007; Gulati et al. 2021). One carer suggested that accurately documenting food intake can be challenging because residents may take food away with them, or they may not remember what they have eaten. Conversely, carers also believed it was important to foster residents' independence by not closely monitoring their food intake. Instead, they found it more beneficial to help residents establish a routine of consuming three meals a day without constant reminders, as residents will ultimately need to develop this ability independently.

Carers engaged in discussions regarding their approach to promoting healthy eating and the extent of their influence on their clients' food choices. While some carers believed in restricting their clients' access to unhealthy foods for their overall well‐being, others placed a strong emphasis on respecting clients' right to autonomy and decision‐making. Some carers recognized the delicate balance between promoting healthy eating and honouring clients' autonomy. They described their efforts to educate clients about the advantages of healthy eating and encourage them to make improved food choices. However, they also acknowledged that they could not impose food restrictions or dictate clients' eating preferences; instead, they had to honour and respect clients' decisions:

we would try to offer a healthier option and I would say ‘you know this is good for you it's healthy’. … we have to let them make their own choice. I would carry on encouraging them to eat the healthier option and explaining the benefits of eating, if they could understand it … I would try to do as much as I could and offering different healthy things … If they say no, maybe next time try again with something else and see. You never know just keep trying … persevere.

The challenge lies in finding a harmonious equilibrium between autonomy and protection while considering external factors such as differing perspectives from family members regarding appropriate care. Ideally, families and carers should work collaboratively to ensure that clients' needs and preferences are met, and they receive comprehensive care and support. Effective communication and mutual respect are pivotal in building and sustaining a positive relationship between families and carers.

iii Opportunities for working together to support dietary choices

This theme highlights the emphasis on providing praise, support, and advice to encourage healthier eating habits, while also acknowledging the constraints imposed by regulations and individual autonomy. The findings from our study, based on interview data, indicate that carers recognised the impact of various behavioural strategies, including positive reinforcement, on clients' eating patterns:

She likes a lot of praise – so the more you praise her if she is eating healthily and she is losing the weight… … We have given her certificates that we have printed off and just mocked up on the computer very quickly. She loves that kind of thing because she just loves the praise that she has done something that was good because historically in her childhood she never got that. She never got any praise for doing anything good.

They emphasised that encouraging clients to maintain healthy behaviours is an effective approach (Epstein et al. 2007). Research conducted by Overwijk et al. (Overwijk et al. 2022) supports the use of behaviour modification strategies to promote healthy lifestyles in individuals with intellectual disabilities. These strategies include providing feedback on performance, giving instructions on behaviour execution, engaging in activities together, rewarding successful behaviour, acknowledging effort towards behaviour change, modifying the environment, implementing graded tasks, using prompts for practice, and modelling/demonstrating behaviour (Spanos, Hankey, et al. 2013).

One more approach highlighted in our study is engaging in negotiations. Coercing individuals into adopting healthy behaviours was seen to be ineffective. Regardless of the positive or negative nature of the behaviour, forcing someone to do something may not yield the best outcomes:

That's their choice and there is nothing we can do other than say ‘you have been out this week, you have bought your snacks for the week and when they are gone they are gone’. If it is gone in a day well that is up to them. Yes, we do have issues with a lot of overeating ……. There is nothing really that we can do because in their best interests you can go to a dietitian who will advise try this, this and this and we can go ‘yes OK’ but we can't force them.

A home is a vital component of one's identity, social relationships, independence, and participation (Svanelöv 2020). The notion of a “home” encompasses the principles of autonomy, privacy, and freedom of choice, where individuals are entitled to conduct their own affairs (Svanelöv 2020; Smith 1994). However, support provided in group homes is often based on staff members' knowledge of residents' needs and experiences, which can limit residents' involvement and undermine their input in day‐to‐day decisions (Svanelöv 2020; Altermark 2017). While staff members are present in residents' homes to promote engagement and independence, it is important to avoid approaches that resemble disciplining or coercing residents into compliance (Svanelöv 2020; Hellzen et al. 2018). Our study demonstrates that a more positive and engaging approach tends to evolve in such settings.

Shared decision‐making, a concept not limited to medical care, involves healthcare professionals or carers providing necessary information about options to clients, who then express their preferences and values before a decision is made (Stiggelbout et al. 2012). While shared decision‐making has been associated with higher service user satisfaction (Shay and Lafata 2015), it becomes more complex when working with individuals who may face barriers to actively participating in conversations, such as those with intellectual disabilities (Noorlandt et al. 2021).

The Committee on the Rights of Persons with Disabilities (CRPD) prompted a shift towards considering “the will and preferences” rather than relying solely on the “best interests” approach. This change aims to ensure that people with disabilities can exercise their right to legal capacity on an equal basis with others (Bigby et al. 2022). Respecting the right to participate in decision making and being mindful of will and preferences helps safeguard against substitute decision making and undue influence from others. However, variation in respecting personal preferences can arise due to the type of decision, the context, and the beliefs and values held by the carer, leading to actions that range from controlling to enabling (Browning et al. 2021).

The use of semi‐structured in‐depth interviews with carers, enriched with real‐world examples of challenges and issues, strengthens the study by providing a comprehensive understanding of food‐related practices and dietary support for individuals with intellectual disabilities. Despite these strengths, this study has limitations that should be acknowledged. The study was conducted within a specific geographic region and cultural context, which may not fully capture the experiences and practices of carers in other regions or cultures with different food traditions and healthcare systems. Additionally, detailed information on the hours of support provided per week for individual clients or systematic categorization of the levels of intellectual disability was not collected. Future research could address these gaps to provide a more comprehensive analysis of the context in diverse care settings.

Carers and managers in residential homes hold a significant position to influence the diet and lifestyle choices of the residents. However, there is a need to improve nutritional care in community services and implement evidence‐based measures to promote a healthy diet (Kolset et al. 2018; Murphy 2017; Truesdale and Brown 2017). Many carers working in residential houses receive inadequate education on healthy eating and food preparation skills, despite their potential to influence health behaviours and lifestyle choices (Ptomey and Wittenbrook 2015; Melville et al. 2009). A study in Norway highlights that training carers in nutritional knowledge and involving nutritionists can significantly improve dietary habits and weight management among individuals with intellectual disabilities in group homes (Gordeladze et al. 2022). However, most health promotion efforts for this population remain predominantly individual‐focused and less effective compared to broader interventions targeting environmental and socio‐ecological factors (Dean et al. 2021). This underscores the importance of integrating intellectual disabilities into nutrition and obesity prevention policies, addressing the broader food environment, and providing comprehensive nutrition education for carers to achieve better nutritional outcomes. Additionally, differing views among carers and managers on their roles in promoting a healthy lifestyle have been observed (Bergström and Wihlman 2011) and present an opportunity to collaborate with nutritionists to enhance health support initiatives in these settings and underscore the significance of this paper. There is also an opportunity for nutritionists and dietitians working in weight management services with this client group to involve carers or family in discussions around strategies to create supportive achievable solutions that can be incorporated into real‐world care settings and therefore the findings have applicability to future practice. Finally, we recommend the inclusion of bespoke nutrition training as part of the standard training provided to carers in residential and family settings, particularly during the early years when dietary habits are being established. Such training should encompass knowledge of food groups, their relationship to for example the Eatwell Guide (UK) or other country level guides, and practical guidance on how to apply this to shopping and meal choices. It should also cover approaches to weight management, including evidence‐based strategies for weight loss and the types of support available. Furthermore, carers should be equipped with an understanding of special dietary requirements and how to promote adherence while accommodating individual needs. Whilst these are some ideas that came from research discussions, we would suggest the co‐creation of an appropriate training course with carers based on their needs.

## 4 Conclusion

This study provides valuable and significant insights into the dietary practices and challenges faced by carers supporting individuals with intellectual disabilities. Our findings highlight the critical role of carers in mediating between individual preferences, nutritional needs, and external constraints and the need for training to better enable carers to support their clients.

To improve nutritional care for people with intellectual disabilities, future research should focus on co‐designing and evaluating the effectiveness of nutrition training for carers and exploring the role of nutritionists/dietitians in supporting families. Policies should integrate the specific needs and challenges of those with intellectual disabilities into broader nutrition and obesity prevention strategies and improvemnt approaches should, emphasise the need for comprehensive training for carers in both residential and family settings. Additionally, practical recommendations include promoting collaborative approaches between carers and dietitians to create achievable, sustainable dietary changes.

## Author Contributions

A.Ö. and B.E.: study conceptualisation. R.H. and B.E.: data collection. A.Ö., B.E., R.H., H.C.: data analysis. A.Ö., B.E. and H.C.: paper writing. All authors approved the final manuscript.

## Funding

This research was supported with funding from the University of Chester QR funds (QR 225).

## Ethics Statement

Ethical approval was received from the Faculty of Health and Social Care Research ethics sub‐committee.

## Conflicts of Interest

The authors declare no conflicts of interest.

## Supporting information


**Table S1:** Theme development scheme.

## Data Availability

The data that support the findings of this study are available from the corresponding author upon reasonable request.
