# Supplementary material for: Eating Well When Living With an Intellectual Disability—Exploring the Carer: Client Relationship in Residential Settings
Source: J Appl Res Intellect Disabil. 2025 Nov 30;38(6):e70157. doi: 10.1111/jar.70157 (PMC12665284; doi:10.1111/jar.70157)
Supplement: Supplementary file 1 — Table S1: Theme development scheme. [file JAR-38-e70157-s001.docx]

**Interview schedule**

Healthy diets

What do you think makes up a healthy diet?

Why is a healthy diet important?

Do you think people you care for have a healthy diet? Explore answer.

Do you think your clients’ health is influenced by poor diet?

Responsibility

Whose responsibility is it to ensure the client gets a healthy diet and why? Explore answer?

Typical diet history

Describe the typical diet of a person you care for? Is that different to your own? If so, how does it differ?

Why are these foods eaten?

The food chain

Describe how food is accessed: purchasing foods and preparation/cooking

How are choices about meals, weekly menu and shopping lists made?

Are there any restrictions to eating patterns e.g. provided by central facility or times of day food can be eaten etc.

What financial considerations do you need to bear in mind?

Choices

What can you do in practice to influence the diet of your clients?

What do you do?

If clients have preferences which are unhealthy – what do you do?

What are your views about food being utilized as a means of reinforcement or a reward for positive behaviour?

**Table S1.** Theme Development Scheme

| **Original Theme** | **Initial Codes/Sub-Codes** | **Evolved Concept** | **Developed Theme** |
| --- | --- | --- | --- |
| Influence on clients’ diets | - Responsibility on clients’ diet  - Carers’ influence on diets  - Carers' attitudes (advise, praise, restrict, encourage)  - Monitoring  - Families' behaviours | From influencing choices and control to a shared responsibility dialogue, ethics, and blurred boundaries  → On-going dialogue to influence and guide around choices  → Delivering the personal plan for high dependency adults  → Responsibility and duty of care to ensure a healthy diet?  → Respecting clients’ choices  → Blurred boundaries – Challenges of advice and enforcement | **Whose responsibility is it?** |
| Dietary habits | - Clients’ dietary choices  - Families' behaviours  - Less dependency  - Challenging behaviours (food stashing)  - Clients getting involved in food process | From individual choice to exploring autonomy within relationships and influence dynamics  → Food as a reward  → Less dependency  → Families as external influences on eating – challenges/barriers (and opportunities) | **Food autonomy and choice in the context of caring relationships** |
| Struggle with disabilities |  |  |  |
| Food process |  |  |  |
| Influence on clients’ diets | - Praise, encouragement, advice  - Monitoring diets  - Support from dietitians/nutritionists  - Clients involved in food preparation  - Negotiating food choices, balancing autonomy with health goals | Reframing these actions into collaborative strategies and shared efforts to support dietary choices  → Positive reinforcement and practices for healthy behaviours  → Negotiations around behavioural choices  → Providing advice | **Opportunities for working together to support dietary choices** |
| Food process |  |  |  |
| Influence on clients’ diets |  |  |  |
